# Shared mechanism for emotion processing in adolescents with and without autism

**DOI:** 10.1038/srep42696

**Published:** 2017-02-20

**Authors:** Christina Ioannou, Marwa El Zein, Valentin Wyart, Isabelle Scheid, Frédérique Amsellem, Richard Delorme, Coralie Chevallier, Julie Grèzes

**Affiliations:** 1 Laboratoire de Neurosciences Cognitives, Inserm unit 960, Département d’Etudes Cognitives, Ecole Normale Supérieure, PSL Research University, Paris, 75005, France; 2 Centre Expert Asperger, Fondation Fondamental, Paris, 75019, France; 3 Service de Psychiatrie de l’Enfant et de l’Adolescent, Hôpital Universitaire Robert Debré, Paris, 75019, France; 4 Génétique Humaine et Fonction Cognitive, Institut Pasteur, Paris, 75015, France

## Abstract

Although, the quest to understand emotional processing in individuals with Autism Spectrum Disorders (ASD) has led to an impressive number of studies, the picture that emerges from this research remains inconsistent. Some studies find that Typically Developing (TD) individuals outperform those with ASD in emotion recognition tasks, others find no such difference. In this paper, we move beyond focusing on potential group differences in behaviour to answer what we believe is a more pressing question: do individuals with ASD use the same *mechanisms* to process emotional cues? To this end, we rely on model-based analyses of participants’ accuracy during an emotion categorisation task in which displays of anger and fear are paired with direct *vs*. averted gaze. Behavioural data of 20 ASD and 20 TD adolescents revealed that the ASD group displayed lower overall performance. Yet, gaze direction had a similar impact on emotion categorisation in both groups, *i.e*. improved accuracy for salient combinations (anger-direct, fear-averted). Critically, computational modelling of participants’ behaviour reveals that the same mechanism, *i.e*. increased perceptual sensitivity, underlies the contextual impact of gaze in both groups. We discuss the specific experimental conditions that may favour emotion processing and the automatic integration of contextual information in ASD.

Autism spectrum disorders (ASD) are neurodevelopmental conditions characterised by significant deficits in social interaction and communication skills, associated with repetitive and restricted interests^1^. Atypicalities in the affective domain are central to ASD and research suggests that individuals with ASD react to social cues differently from typically developing (TD) individuals^2^,^3^,^4^. The roots of such difficulties are still debated but it has been suggested that difficulties in processing emotional cues^3^,^4^,^5^ play an important role in these social deficits. This hypothesis has led to a considerable amount of work that is partly synthesised in a meta-analysis of 48 studies involving nearly one thousand participants with ASD^3^. Overall, research points to emotion recognition difficulties and to reduced activation of emotion related brain areas in autism^6^,^7^. However, a number of studies (including ones with large sample sizes and well-matched groups) have found that people with ASD do recognise emotions accurately^8^,^9^,^10^. In this paper, we shift the focus to ask whether the *mechanisms* behind the processing of threat-related emotional expressions (anger/fear) are the same in ASD, irrespective of potential differences in accuracy between ASD and TD groups. To do so, we focus on the contextual impact of gaze direction on emotion recognition because it is theoretically possible to distinguish different mechanisms that may affect the integration of these social cues^11^.

Emotional displays are often ambiguous and the context in which they are presented also informs emotional decoding. For instance, recognition of threat-related emotional expressions is often informed by gaze direction, especially in cases where the expression is ambiguous^12^,^13^,^14^,^15^,^16^,^17^: TD individuals are more likely to judge a neutral face as angry when presented with a direct gaze and as fearful when presented with an averted gaze^14^. TD individuals are also quicker and more accurate to recognise anger presented with a direct gaze because it signals to the observer that they are under imminent threat, and fear with an averted gaze because it signals a potential threat in their surroundings^16^. These combinations of gaze direction and emotional expressions are thus more salient to the observer^11^ compared to the reverse combinations (anger with averted and fear with direct gaze). Up until recently, the mechanisms through which these phenomena occurred remained unknown. Indeed, classical decision theory distinguishes two manners in which gaze direction might influence emotion categorisation: through a change in decision bias toward highly salient threat-signalling combinations or through enhanced perceptual sensitivity to these combinations. Recent work^11^ suggests that improved decoding of specific combinations of gaze and emotion is associated with a selective enhancement of perceptual sensitivity in TD adults.

In ASD, it is unclear whether the impact of gaze direction on emotion categorisation is intact and whether the mechanisms they rely on are the same as those used by neurotypicals. Existing data suggest that highly salient threat combinations (anger-direct, fear-averted) are not recognized more quickly than less salient combinations in ASD and that they fail to elicit larger face-sensitive event related potentials (ERP) in ASD children^18^,^19^. Similar results were also found in ASD adults, using fMRI^20^. Taken together, these findings suggest that individuals with ASD may use different mechanisms to combine contextual information, specifically gaze direction, with emotional expressions of anger and fear when categorising emotions. However, a number of issues limit the scope and generalizability of these findings. Specifically, in these studies, the emotional expressions used were stereotypical, of high intensity, and of long duration. Yet, the automatic impact of gaze direction on emotion perception takes place in the brain within 200 ms after stimulus onset, for short stimuli presentation (<300 ms) and, is more prominent when the expression is ambiguous and hence more difficult to discriminate^11^,^21^,^22^. Further, with the exception of one study^20^, participants were not explicitly instructed to fixate the eye-region of the face despite evidence linking attention directed to the eye-region and emotion recognition performance^23^. Since ASD individuals do not spontaneously orient to the eye region^24^,^25^,^26^, the lack of explicit instruction may have put the ASD group at a disadvantage^26^,^27^,^28^.

Given the issues reported above, the goal of the present experiment was twofold. First, we aimed to determine whether participants with ASD use gaze signals to inform emotional decoding of anger and fear under well-controlled experimental conditions. To do so, we adapted an emotion categorisation task^11^ controlling for a range of potential confounds: emotion intensity was manipulated parametrically across seven levels of “morphed” facial expressions ranging from neutral to intense anger or fear; contextual information was included by pairing facial expressions with direct or averted gaze; participants’ attention was drawn to the eyes of the upcoming face by presenting a fixation cross right before the appearance of the stimulus; finally, faces were flashed for 250 ms in order to tap into the automatic (reflexive rather than reflective) stage of processing^22^. Our second goal was to determine whether, under such experimental conditions, the mechanisms behind threat-related emotion-gaze integration are similar in TD and ASD individuals.

## Results

**Figure 1:**
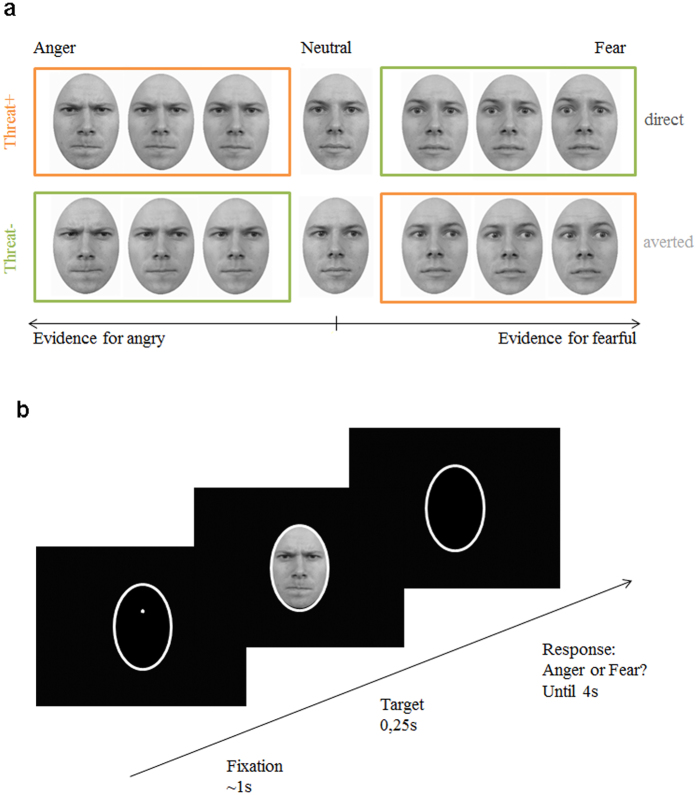
Stimuli and experimental procedure. (**a**) Example of facial expressions morphed parametrically from neutral to intense fearful/angry expressions providing evidence for one or the other emotion. Each face was either paired with a direct or an averted gaze. Threat+ conditions (in orange) correspond to combinations of gaze and emotion that signal higher salience and threat for the observer as compared to Threat− conditions (in green). (**b**) For each trial, and following a fixation (1 sec), a face appeared for 250 ms, and participants had a 4 second response window to indicate whether the face expressed fear or anger.

The experimental task was a two-choice emotion categorisation task (fear or anger). In each trial participants were presented with a facial expression of anger or fear of varying intensity (7 levels of emotion strength), paired with direct or averted gaze, and had to categorise the expressed emotion (see Fig. 1). The concomitant gaze direction was not mentioned to the participants and hence was implicit.

Participants completed 3 blocks of 120 trials. We first ran an ANOVA including blocks as a factor to investigate potential effects and interaction with blocks. An effect of blocks (F(2,37) = 3.795, p = 0.032, 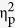 = 0.170) revealed that participants’ emotion accuracy increased over time with significantly better performance in the third block (85%) as compared to the first (80%). No other effects or interactions were significant, so block was not included as a factor in the remaining analysis. Overall, both groups performed above chance (ASD Median = 82%, Z = 3.920, p < 0.001, r = 0.62; TD Median = 88%, Z = 3.920, p < 0.001, r = 0.62) although adolescents with ASD reached a significantly lower mean accuracy level (82%) than TD adolescents (86%) (Effect of group: F(1,38) = 4.479, p = 0.041, 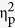 = 0.105).

### Increased accuracy with emotion strength in both TD and ASD adolescents

Categorisation performance of both groups increased with emotion intensity (F(6,228) = 53.745, p < 0.001, 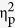 = 0.706). An emotion by intensity interaction (F(6,228) = 12.433, p < 0.001, 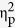 = 0.247) led to enhanced categorisation performance with emotion strength for both anger (Effect of intensity on anger: F(6,228) = 54.952, p < 0.001, 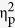 = 0.591) and fear (Effect of intensity on fear: F(6,228) = 13.267, p < 0.001, 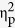 = 0.295).

### Overall emotion and gaze direction effects

Both groups showed enhanced recognition of fear (ASD: 86%; TD: 89%) in comparison to anger (ASD: 78%; TD: 84%) (Effect of emotion; F(1,38) = 10.625, p = 0.002, 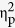 = 0.222; no emotion by group interaction: F(1,38) = 0.424, p = 0.519, 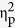 = 0.011). One possible explanation for this fear advantage is related to an enhanced saliency of fear signals, which are perceived as instinctive reactions to an overall threatening environment^29^. This is suggested by a recent paper^29^ in which the authors compared search efficiency for angry and fearful expressions, both being negative emotions signaling danger, embedded in a crowd of neutral faces. Contrary to the anger-superiority hypothesis, they found better performance for fearful faces, as in the present study. The authors proposed that while anger signals a direct-threat (and therefore an unambiguous source of threat), fearful faces signal an indirect and more diffuse threat, and are therefore more salient.

The only difference in performance between the two groups was that the TD adolescents performed better overall in direct gaze conditions (Z = 3.659, p < 0.001, r = 0.58) compared to averted gaze conditions, while emotion accuracy in the ASD group did not differ between the two (Z = 1.493, p = 0.135, r = 0.24; Gaze * Group interaction: F(1,38) = 5.263, p = 0.027, 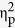 = 0.122). This is consistent with previous research demonstrating an overall advantage of TD individuals in direct gaze conditions^30^,^31^,^32^.

### Increased recognition of threatening conditions in both TD and ASD adolescents

**Figure 2:**
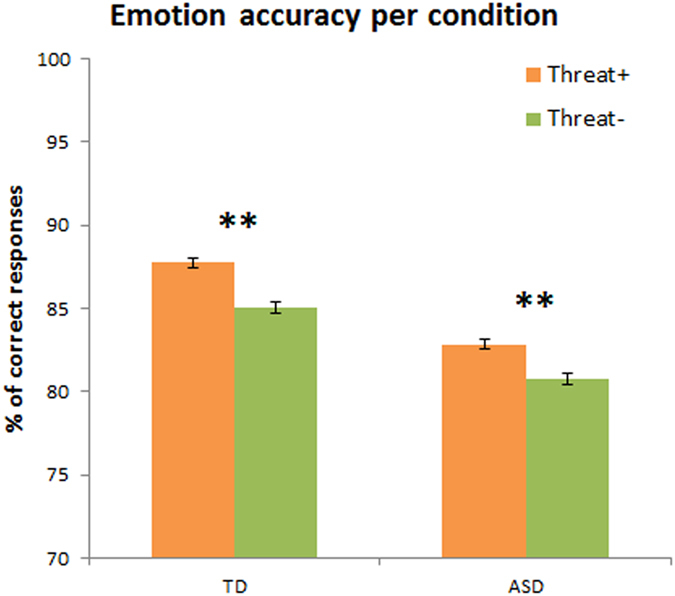
Emotion accuracy results for Threat+ and Threat− conditions for the TD group and the ASD group. Threat+ combinations were recognised more accurately than Threat− ones in both groups. Note that there was a main effect of group with the TD group demonstrating overall higher emotion recognition accuracy than the ASD group, but no interaction between group and Threat conditions. Within subject error bars represent Mean ± S.E.; *p < 0.05, **p < 0.01.

Contrary to previous studies in ASD^18^,^19^, this study replicates in TD and ASD adolescents previous findings^11^ in adults of an influence of contextual gaze direction on categorisation of threatening emotions (Emotion * Gaze: F(1,38) = 26.242, p < 0.001, 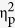 = 0.408). We observed higher accuracy in the recognition of angry facial expressions when associated with a direct gaze as compared to an averted gaze, and higher accuracy in the recognition of fearful faces when associated with an averted gaze as compared to a direct gaze. Importantly, the present experiment reveals that this interaction did not differ between groups (Emotion * Gaze * Group: F(1,38) = 0.287, p = 0.595, 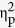 = 0.007). Indeed, within group analyses revealed that the Emotion by Gaze interaction was significant in both the TD (F(1,19) = 16.373, p = 0.001, 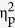 = 0.463) and the ASD group (F(1,19) = 10.291, p = 0.005, 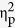 = 0.351) with both groups identifying highly salient threat combinations (anger-direct, fear-averted, labelled Threat+) better than less salient combinations (anger-averted, fear-direct, labelled Threat−; TD: Z = 2.931, p = 0.003, r = 0.46; ASD: Z = 2.696, p = 0.007, r = 0.42; see Fig. 2). Moreover, the variance of accuracy scores in Threat+ and Threat− were homogenous across the two groups (Threat+: F(1,38) = 2.313, p = 0.137; Threat−: F(1,38) = 0.537, p = 0.468).

Additionally, we computed the Bayes factor to test for the strength of the difference between Threat+ and Threat− in ASD as compared to TD^33^ (see method section). We obtained a Bayes factor higher than 3 (Bayes factor = 32) when comparing the difference between Threat+ and Threat− in TD and ASD, confirming an increase in accuracy for Threat+ conditions in ASD group, similarly to the TD group.

### Mechanisms underlying increased recognition of threatening conditions

**Figure 3:**
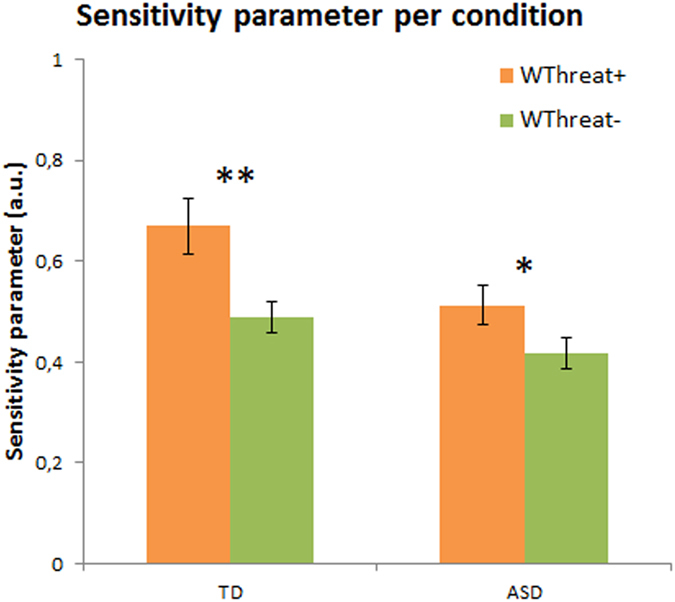
Perceptual sensitivity parameter estimate for Threat+ and Threat− combinations for the TD group and the ASD group. Both groups showed enhanced perceptual sensitivity for highly salient emotion-gaze combinations (Threat+). Error bars represent Mean ± S.E.M.; *p < 0.05, **p < 0.01.

To assess whether we replicate the increased perceptual sensitivity to threatening emotions found in healthy adults^11^, we compared different models that could explain the participants’ behaviour. In the framework of Signal Detection Theory^34^, an increased performance for Threat+ conditions could either stem from a *decision bias* toward these conditions (model 1), or from an increase in the *perceptual sensitivity* to these combinations (model 2) (see methods for details on the models and model comparisons). Fixed-effect Bayesian model selection (Bayesian information criterion) showed that an increased sensitivity to Threat+ conditions explained the data better than a change in the decision bias, in both TD and ASD groups (TD: Bayes Factor ≈ 93, ASD: Bayes Factor ≈ 80). The sensitivity parameter estimate was significantly enhanced for Threat+ conditions as compared to Threat− in both the TD (Fig. 3; p < 0.01, standardized effect size = 2.7, see Methods for details) and the ASD (Fig. 3; p = 0.03, standardized effect size = 1.7, see Method for details) group (see Fig. 3).

### Reaction time analyses

Although TD adolescents (M = 350.8, S.E.M. = 5.86) had faster general RTs (assessed by the Go/no-Go task) in comparison to the ASD group (M = 380.4, S.E.M. = 9.98; U = 115.000, ASD = TD = 20, p = 0.021, r = 0.36), to our surprise, ASD participants were quicker at responding during the emotion categorisation task as compared to the TD group (Effect of group: F(1,38) = 13.819, p < 0.001, 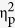 = 0.267). Previous findings on RTs in ASD are mixed: some studies find that these individuals take longer than TD individuals to complete emotion categorisation tasks^35^, others find no difference between the two groups^18^,^36^, while still others, using several different visual search or detection tasks^37^,^38^, report the opposite, finding ASD individuals quicker than TD individuals. Yet, it has been generally suggested that in individuals with ASD, better visual search is associated with shorter RTs^38^. Still, given that ASD showed faster general RTs associated with a decreased general performance, a speed-accuracy trade-off effect may be happening. We however believe that our main result, i.e. increased performance for Threat+ as compared to Threat− (interaction between gaze and emotion) cannot be explained by such potential speed-accuracy trade-off, as there was no significant interaction between gaze and emotion on RTs (F(1,38) = 0.595, p = 0.445, 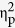 = 0.015; TD F(1,19) = 0.192, p = 0.666, 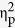 = 0.01; ASD F(1,19) = 3.914, p = 0.063, 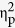 = 0.171). Furthermore, we checked whether there was a correlation between general performance and RTs in ASD as a speed-accuracy trade-off proxy: a regression analysis revealed that mean RTs is not a significant predictor of ASD participants’ mean performance (F(1,19) = 1.070, p = 0.315).

### Gaze direction effects

We conducted RTs analyses to examine whether gaze direction has an influence on the speed with which participants decode the two emotions. Firstly, both TD and ASD adolescents’ became quicker in responding when the expressed emotions were more intense, as reflected by the intensity by group interaction (F(6,228) = 5.364, p < 0.001, 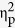 = 0.124; F(6,114) = 6.286, p = 0.002, 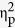 = 0.729; ASD F(6,114) = 6.558, p = 0.002, 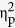 = 0.338).

Secondly, in both groups, emotions presented with direct gaze were identified quicker than emotions coupled with averted gaze (F(1,38) = 7.317, p = 0.01, 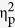 = 0.161). This could be linked to several previous findings: 1) direct as compared to averted gaze is generally easier to detect^39^; 2) direct gaze leads to quicker RTs regardless of it being presented as part of a face or in isolation^40^ and; 3) both ASD and TD individuals detect quicker targets with direct gaze than targets with averted gaze^41^.

Moreover, there was a Gaze * Group interaction (F(1,38) = 4.171, p = 0.048, 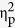 = 0.099): ASD participants’ RTs were comparable between direct and averted gaze (Z = 0.747, p = 0.455, r = 0.12) while the TD participants’ RTs were quicker for direct gaze conditions as compared to averted gaze conditions (Z = 2.501, p = 0.012, r = 0.39), a result further confirming the better overall performance of TD individuals when the gaze is directed towards them in the present study.

### Controlling for baseline RTs difference between group

Finally, in an attempt to control for potential baseline reaction time differences, we also ran our analyses while co-varying Go/no-Go RTs out. We found the same pattern of results: an effect of group (F(1,37) = 17.986, p < 0.001, 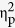 = 0.327), no interaction between gaze and emotion (F(1,37) = 1.567, p = 0.219, 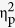 = 0.041; TD F(1,18) = 0.273, p = 0.608, 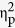 = 0.015; ASD F(1,18) = 1.502, p = 0.165, 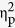 = 0.104). There was an intensity * group interaction (F(6,222) = 5.066, p = 0.003, 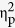 = 0.120; TD F(6,114) = 23.347, p < 0.001, 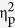 = 0.551; ASD F(6,114) = 10.081, p < 0.001, 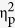 = 0.347), an effect of gaze (F(1,37) = 5.779, p = 0.021, 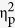 = 0.135), with emotions with direct gaze better identified and this as a function of participants’ general reaction times (Gaze * RTs general interaction, F(1,37) = 7.293, p = 0.010, 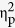 = 0.165) and finally, a gaze * group interaction (F(1,37) = 9.426, p = 0.004, 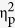 = 0.203).

To conclude, following the absence of a significant interaction between emotion and gaze direction on RTs in both groups, our results did not replicate the speed advantage for categorising Threat+ as compared to Threat− conditions previously demonstrated in TD adolescents^18^ and neurotypical adults^11^.

## Discussion

The present experiment aimed to determine whether the mechanisms behind emotion-gaze integration are similar in TD and ASD individuals, irrespective of potential group differences in accuracy. The results show that adolescents with ASD, similarly to TD controls, are more accurate when decoding highly salient combinations of gaze and emotion, demonstrating that they combine task-unrelated gaze information with emotion. Importantly, although TD participants had higher overall recognition accuracy than ASD participants, the fitting of decision theoretical models to the behavioural data revealed that, in both TD and ASD adolescents, gaze direction enhanced perceptual sensitivity to highly salient combinations, resulting in the associated improved accuracy.

These results stand in sharp contrast with previous observations^18^,^19^,^20^ showing that contextual gaze direction has little impact on emotion categorisation in ASD. However, our experimental set-up differs in several important ways. First, the ambiguity of sensory evidence was manipulated using graduated morphs moving from neutral to angry or fearful expressions. This is important because the impact of gaze direction is particularly clear in ambiguous situations where emotion discriminability is difficult^11^,^42^. Second, the contextual cue (gaze direction) co-occurred with the decision-relevant stimulus but was irrelevant to the emotion categorization task, and thus did not need to be processed explicitly. Third, the facial expressions were presented for a very limited period of time, which allowed us to specifically tap into automatic decoding processes^22^. Finally, participants’ attention was drawn to the eye region by displaying a pre-stimulus fixation point at the eye-level of the upcoming face stimulus. This feature of the task is particularly decisive for ASD participants who do not preferentially attend to social stimuli^24^,^43^, such as the eyes^24^,^25^,^26^, and may be at a disadvantage^26^,^27^,^28^ in emotion categorisation tasks where attention is not expressly drawn to them. Indeed, emotion recognition performance is positively related to attention to the eye-region^23^, notably for negative emotions such as fearful and angry expressions, primarily expressed using the upper part of the face^44^.

Under the specific experimental conditions used in our design, we found that gaze direction has a similar impact on performance in emotion categorisation in ASD and TD participants. Although overall performance was higher in the TD group, individuals with ASD were able to integrate co-emitted social signals of gaze and emotional expression to inform emotion decoding. A key innovation of this study was to reveal the mechanism that instantiates the contextual impact of gaze direction on emotion categorisation by fitting theoretical decision models to the behavioural data. It is indeed conceivable that participants with ASD reach a higher level of performance in the salient gaze-emotion conditions by resorting to underlying processes that are completely different from those used by control participants. Critically, we found that the same mechanism was at play in the ASD and in the TD groups and that improved recognition accuracy for highly salient threat-signalling emotion-gaze combinations corresponded to a selective enhancement of perceptual sensitivity to these combinations of gaze and emotion. Thus, the present findings demonstrate that ASD adolescents are not only able to decode emotions but that they also automatically integrate contextual gaze while doing so.

The present study thus extends previous evidence of intact face processing^45^ and intact prioritisation of salient social cues over less salient ones^46^ in ASD, by demonstrating that adolescents with ASD rely on the same mechanism as TD adolescents to combine contextual social cues (here gaze direction) with facial displays of emotions as a function of their salience for the observer. Given that the brief and sudden fixation point at the eye region (which triggers attention) was one of the critical differences between the current study and previous studies where participants with ASD failed to integrate social cues, it is possible that diminished spontaneous attention to the eyes accounts for at least part of the atypicalities in emotion processing commonly reported in ASD. This view is also compatible with the idea that diminished automatic orientation to socially relevant signals is a core deficit in ASD^28^ as well as with data demonstrating that the processing of socially relevant signals is intact under motivated conditions^47^. Future work will therefore need to manipulate eye fixation directly in order to confirm whether this is indeed a key parameter guiding emotion categorisation in ASD. Since our sample size is relatively small, it will also be important to replicate this work and assess its generalizability to various subtypes of ASD. However, it is important to note that the effect we report here replicates what has already been described in a sample of 24 healthy adults^11^, which suggests that our effect is robust.

To conclude, our results demonstrate not only that adolescents with ASD take into account contextual gaze information while processing emotional displays, but more importantly, even though their overall emotion recognition accuracy is lower than TD adolescents, that the same mechanism, *i.e*. increased perceptual sensitivity, underlies such contextual impact in both groups. These results suggest the possibility that significant difficulties in social interaction and communication seen in ASD may exist independently of their ability to process the social signals themselves. Future experiments should address whether, when decoding skills appear preserved, ASD’s social difficulties are related to dysfunctions in the motivation mechanisms driving attention to socially relevant signals or to the mechanisms underlying the preparation of appropriate response behaviour to perceived social signals, both crucial to social interactions in daily life.

## Methods

### Participants

Twenty-four adolescents with ASD aged between 12 and 17 years old and 24 TD adolescents participated in this study. Adolescents in the ASD group were recruited from the University Hospital Robert Debré (Paris, France). Final diagnosis of ASD was based on DSM IV-TR^48^ criteria and made by summing the information from the Autism Diagnostic Interview-Revised (ADI-R)^49^, the Autism Diagnostic Observation Schedule (ADOS)^50^ and data from clinical reports made by experts in the field. ASD participants’ Intelligence Quotient (IQ) was assessed using the full Wechsler Intelligence Scale for Children version IV^51^ (WISC IV). They were also tested for normal visual acuity using the Freiburg Visual Acuity and Contrast Test^52^ (FrACT version 3.8.2). This test was adapted to the distance of 0.3 meters. Normal vision was ensured by a Snellen fraction of 0.3/0.3 (distance of test/distance at which the subject can identify the indicated symbol). Trait anxiety was assessed using an abbreviated form^53^ of the State-Trait Anxiety Inventory^54^ (STAI; See Table 1). Finally, no participants were on medication during the period of the study.

Adolescents in the TD group were recruited from a mainstream school. They did not report any history of developmental or other psychiatric illness. They all had normal or corrected to normal vision. Due to time constraints, IQ in the TD group was assessed with the French Wechsler Abbreviated Scale of Intelligence (four subsets) which has been found to be highly reliable in giving a representative score of the full IQ^55^ in the general population (See Table 1).

The experimental protocol and associated methods were approved by INSERM and licensed by the local research ethics committee (ClinicalTrials.gov Identifier: NCT02628808, Protocol Study ID: 2008-A00019-46). Our study was performed in accordance with the Declaration of Helsinki. All parents and children provided written informed consent according to institutional guidelines of the local research ethics committee. All the participants were debriefed and thanked following their participation.

### Materials and design

The stimuli consisted of 12 face identities (6 female) selected from the original set^11^ of 36 identities. Two (1 female) additional identities were used during training only. The original identities were drawn from the Radboud Face Database^56^ and were modified^11^ using Adobe Photoshop CS5.1 (Adobe Systems, San Jose CA) and parametrically morphed^11^ using FantaMorph (Abrosoft http://www.fantamorph.com/) so that, for each identity, 30 stimuli were created; 7 morphs (emotion intensities) * 2 emotions (fear/anger) * 2 gaze directions (direct/averted), plus two neutral stimuli, one with direct and one with averted gaze. Hence, our task included 360 trials, one third of original number of trials^11^, divided into 3 blocks in order to avoid tiredness and inattention effects.

### Emotion Categorisation Task

The experimental task was a two-choice emotion categorisation task (fear or anger). The stimuli were projected on a black background using Psychophysics-3 Toolbox^57^,^58^ of Matlab (version R2014a) software (http://uk.mathworks.com/). In each trial, participants saw a white oval line that remained throughout the trial to indicate the size and location of the upcoming stimulus. After 500 ms of the oval’s appearance a fixation point appeared at the level of the stimulus’ eyes for 1000 ms, followed by a target face presented for 250 ms. As soon as the face disappeared the participants had a 4000 ms response window to indicate if they thought the face expressed anger or fear. To do so, they pressed one of the two control buttons (Ctrl) on the keyboard. One button represented fear and the other anger. The side of the button corresponding to fear or anger (e.g., Left Ctrl: anger, Right Ctrl: fear) was consistent across trials for each participant but counterbalanced between participants.

### Go/no-Go Task

To control for potential overall RT differences between the two groups we measured their general reaction times (RTs). We systematically performed all RTs analyses without and with co-varying the general RTs out, and found that the results remain the same. Participants took part in a Go/no-Go task, created on E-prime stimulus presentation software (http://www.pstnet.com/eprime.cfm). They saw a white fixation cross on a grey background, in the centre of the screen, followed 67% of the time by a black dot. When the black dot appeared participants had to press the SPACE button as fast as possible.

### Procedure

Participants were seated at 30 cm distance from the laptop. During the Emotion categorisation task, they were told that they would see faces at the centre of the screen and had to indicate whether they thought that the face expressed anger or fear by pressing the corresponding button. Initially, they completed 10 trials of the emotion categorisation task (training), in order to familiarise with the task. In order to avoid boredom and tiredness effects, the main task was divided in 3 blocks of 120 trials each. At the end of each block, participants could see their percentage of accuracy and speed of responding. Participants completed the first block of the task. Subsequently they did the Go/no-Go test and a second block of the main task. Following that, the participants gave their answers to the Anxiety scale verbally to the experimenter before they completed the third and final block of the emotion categorisation task. Finally, their visual acuity was tested.

### Data Analysis

Four ASD and one TD participants were excluded because they were at chance level during the task (accuracy 40–60%) resulting in 20 ASD participants. These 20 ASD participants were then automatically matched, using R Project for Statistical Computing (www.rproject.org) with 20 TD participants (among 23) according to chronological age, gender and IQ (see Table 1). The matched 20 TD and 20 ASD participants had the same levels of anxiety, which is the most prevalent disorder comorbid with ASD^59^ and has been found to increase one’s sensitivity to social threat^60^ (see Table 1). ADOS scores of the ASD group which were not measured with module 4 (n = 1) were calibrated^61^ and descriptive values can be found in Table 1. Analyses were performed only on valid (response) trials. All non-response trials and all trials with RTs less than 200 ms were excluded from the analysis. Lastly, we compared the total number of non-response trials between the two groups and found no significant differences (see Table 1).

### Model-free analyses on performance

Mean emotion accuracy, as well as the standard error of the mean (S.E.M) for each group are listed in Table 2. RTs descriptive values and results can be found Table 3. Data was analysed using Matlab software and SPSS-18. All p-values reported are two-tailed. Partial eta squared (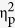) is reported as the effect size of the F statistics, r of the non-parametric comparisons and phi (ϕ) for the chi squared test on gender. A value of 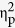 = 0.01/r = 0.1/ϕ = 0.1 represents a small effect size, 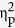 = 0.06/r = 0.3/ϕ = 0.3 a medium one and over 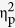 = 0.14/r = 0.5/ϕ = 0.5 a large effect size^62^.

We first performed a 2 × 2 × 7 repeated measures ANOVA on accuracy with Emotion (anger *vs*. fear), Gaze (direct *vs*. averted) and Intensity (7 levels) as within subjects’ factors and Group (ASD *vs*. TD) as a between subject factor. The same analysis was performed within each group independently. As the distribution of the TD group’s residuals was not normal, post-hoc analyses are done using non-parametric statistical tests. In order to compare the performance of each group in highly salient emotion-gaze combinations (Threat+) as compared to less salient emotion-gaze combinations (Threat−), we calculated the mean for each of these two types of combinations.

Further, we calculated the difference between these two means and used it to compute the Bayes factor^63^ for the difference between these two conditions in the ASD group. A Bayes factor uses prior knowledge in association with newly acquired data to describe the likelihood (llh) of the current data in supporting an alternative hypothesis (H1) against a null (H0) and is given by the formula B =  llh_H1_/llh_H0_. If the Bayes factor is above 3 then the data provides support for the H1 while if it is less than 1/3 it provides support for the H0. We wanted to test the H1 that there is a significant difference between Threat+ and Threat− in the ASD group against an H0 that finds no difference between the conditions and for this purpose we used the TD group’s mean difference as the prior.

### Model selection

We used model based analyses to characterise the mechanisms underlying the enhanced performance of the groups in the Threat+ combinations, compared to the Threat− combinations, in the framework of Signal Detection Theory (SDT)^34^. Participants’ behaviour was accounted for using a simple psychometric model:


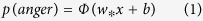


where p(anger) is the probability of selecting the emotion of anger, ϕ is the cumulative normal function and x is the evidence for the corresponding emotion (emotion intensity; from −7 corresponding to intense fear, to +7 corresponding to intense anger, through 0 which represents a neutral expression), *w* to the perceptual sensitivity to the emotional expression (*multiplicative* by the sensory evidence), and *b* to an *additive* stimulus-independent bias toward ‘anger’ or ‘fear’ responses.

We compared two models that could account for the influence of gaze on emotion categorization: model 1, where gaze direction would bias responses towards Threat+ combinations and model 2, where gaze direction would enhance perceptual sensitivity to Threat+ combinations.

A change in the decision bias implies that the bias toward anger or fear is different for direct and averted gaze conditions such as:


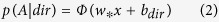



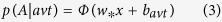


where the probability of selecting the emotion of anger is p(A|dir) in the direct gaze condition and p(A|avt) in the averted gaze condition, *w* is the perceptual sensitivity to the emotional expression (common to all conditions if the effect is on the bias), *b*_dir_ is a bias toward ‘anger’ or ‘fear’ responses in the direct gaze condition, that is different from *b*_avt_, the bias toward ‘anger’ or ‘fear’ responses in the averted gaze condition.

A change on the sensitivity implies that the sensitivity is shared for THREAT+ conditions (Anger direct and fear averted) and different from the sensitivity to THREAT− conditions (Anger averted and fear direct) such as:


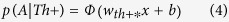



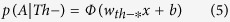


where the probability of selecting the emotion of anger is p(A|Th+) in Threat+ condition and p(A|Th−) in Threat− condition, *w*_th+_ is the perceptual sensitivity to the emotional expression in Threat+ conditions, *w*_th−_ is the perceptual sensitivity to the emotional expression in Threat− conditions, and *b* is a bias toward ‘anger’ or ‘fear’ responses (common to all conditions if the effect is on the sensitivity).

We used Bayesian model selection and calculated the Bayesian information criterion (BIC) to determine which of the two models was more likely to explain the observed data. To check whether TD and ASD participants showed differences in the underlying model best fitting their behaviour (increased performance for Threat+ conditions, which could be due either to changes in sensitivity or decision bias), we applied fixed-effects model comparisons. These comparisons assume that all participants within one group used the same underlying model to generate their behaviour. To compare the two models within each group, we computed the Bayes factor^64^ of the different models as the ratio of each model’s evidence to the compared model’s evidence. To compare sensitivity parameter estimates across Threat+ and Threat− conditions within each group, we computed the marginal posterior probability density function (pdf) of the sensitivity parameter in each condition in 0.01 steps; and computed the empirical probability that the sensitivity parameter in the Threat+ condition is higher than the sensitivity parameter in the Threat− condition (by computing the posterior pdf of the difference in sensitivity parameter between the two conditions, and taking the area under the curve above zero). Importantly, this statistic is independent of the shape of the distribution, but given the approximate Gaussian shape of the difference in sensitivity parameter between conditions, we report standardized effect sizes within each group corresponding to the ratio between the best-fitting mean of the difference divided by the best-fitting standard deviation of the difference (in a least-squares sense).

### Analyses on reaction times

We finally conducted a 2 × 2 × 7 repeated measures ANOVA on reaction times (RTs) with Emotion (anger vs fear), Gaze (direct vs averted) and Intensity (7 levels) as within subjects’ factors and, Group (ASD vs. TD) as a between subject factor (for descriptive values see Table 3).

## Additional Information

**How to cite this article**: Ioannou, C. *et al*. Shared mechanism for emotion processing in adolescents with and without autism. *Sci. Rep.* **7**, 42696; doi: 10.1038/srep42696 (2017).

**Publisher's note:** Springer Nature remains neutral with regard to jurisdictional claims in published maps and institutional affiliations.

## Figures and Tables

**Table 1:** Mean (S.E.M.) of chronological age, gender, total IQ, verbal IQ, performance IQ and trait anxiety for both groups (after automatic matching procedure), total missed trials of each group and ADOS for the ASD group.

	ASD (n = 20)	TD (n = 20)	Test value, p value, effect size value
**Age**	14.10 (0.43)	13.75 (0.33)	U = 178.000, ASD = TD = 20, p = 0.543, r = 0.09
**Gender**	Males n = 16	Males n = 15	Χ^2^(1) = 0.143, p = 0.705, ϕ = 0.06.
**IQ total**	103.2 (4.3)	100.1 (2.1)	U = 184.500, ASD = TD = 20, p = 0.664, r = 0.07
**IQ verbal**	102.9 (5.6)	100.4 (2.2)	U = 189.500, ASD = TD = 20, p = 0.776, r = 0.04
**IQ performance**	104.8 (4.5)	99.65 (2.8)	U = 175.500, ASD = TD = 20, p = 0.507, r = 0.10
**Anxiety**	14.6 (0.9)	13.5 (0.7)	U = 162.000, ASD = TD = 20, p = 0.302, r = 0.16
**Total non-response trials**	32 (0.34)	54 (0.43)	U = 150.500, ASD = TD = 20, p = 0.164, r = 0.22
**ADOS**	11.4 (0.45)	NA	NA

**Table 2:** Mean (S.E.M.) of emotion accuracy per group per condition.

	Conditions:	Anger-Averted	Anger-Direct	Fear-Averted	Fear-Direct	Overall
**ASD**	Accuracy (%)	76 (2)	79 (2)	86 (3)	85 (2)	82 (2)
**TD**	Accuracy (%)	80 (2)	87 (2)	88 (8)	90 (7)	86 (6)

**Table 3:** Mean (S.E.M.) of RTs per group per condition.

	Conditions:	Anger-Averted	Anger-Direct	Fear-Averted	Fear-Direct	Overall
**ASD**	RTs (ms)	866 (38)	841 (36)	855 (40)	869 (38)	858 (37)
**TD**	RTs (ms)	1046 (37)	1021 (33)	1073 (35)	1036 (39)	1044 (34)
